# Efficacy and safety of midazolam versus dexmedetomidine in mechanically ventilated intensive care unit patients: a systematic review and meta-analysis

**DOI:** 10.3389/fphar.2026.1733161

**Published:** 2026-01-28

**Authors:** Wei Peng, Yuan-Yuan Lin, An-Ni Lin, Yong-Jia Zheng, Xu-Liang Cai, Yue-Fei Li, Qian-Yi Tang, Huan He

**Affiliations:** 1 Department of Respiratory and Critical Care Medicine, Guangdong Corps Hospital of People’s Armed Police, Guangzhou, Guangdong, China; 2 Department of Anesthesiology, General Hospital of Southern Theater Command, First Clinical College of Southern Medical University, Guangzhou, Guangdong, China

**Keywords:** dexmedetomidine, intensive care unit, mechanical ventilation, meta-analysis, midazolam, randomized controlled trials

## Abstract

**Background:**

Midazolam and dexmedetomidine are widely used sedatives for mechanically ventilated patients in the intensive care unit (ICU). However, their comparative effectiveness and safety remain debated. This systematic review and meta-analysis aimed to evaluate randomized controlled trials (RCTs) directly comparing these agents.

**Methods:**

The review followed the Preferred Reporting Items for Systematic Reviews and Meta-Analyses (PRISMA) guidelines. PubMed, The Cochrane Library, Web of Science, and Embase were searched through August 2025. Eligible studies were RCTs comparing midazolam with dexmedetomidine in adult ICU patients requiring invasive mechanical ventilation. Outcomes included mechanical ventilation duration, ICU length of stay, delirium, hemodynamic adverse events, and mortality. Pooled estimates were calculated using fixed- or random-effects models, with subgroup and sensitivity analyses performed to assess robustness.

**Results:**

Fifteen RCTs with diverse international populations were included. Dexmedetomidine significantly reduced mechanical ventilation duration (WMD = −0.96 days, 95% CI: −1.56 to −0.36) and lowered delirium risk (RR = 0.59, 95% CI: 0.52–0.68). It was, however, associated with a higher incidence of bradycardia (RR = 2.05, 95% CI: 1.61–2.62). No significant differences were observed in ICU length of stay (WMD = −0.89 days, 95% CI: −2.41 to 0.62) or all-cause mortality (RR = 0.96, 95% CI: 0.79–1.18). Sensitivity analyses confirmed the stability of pooled results. Subgroup analyses suggested stronger benefits of dexmedetomidine in Asian studies and in smaller trials, while the protective effect against delirium was more pronounced in older patient cohorts.

**Conclusion:**

Dexmedetomidine demonstrated clinical advantages over midazolam by reducing delirium and ventilation duration but carried a greater risk of bradycardia. Sedative choice should balance efficacy with cardiovascular safety.

## 1 Introduction

Sedation is an integral component of the management of critically ill patients who require invasive mechanical ventilation in the intensive care unit (ICU). Adequate sedation alleviates patient anxiety, improves tolerance to endotracheal intubation and mechanical support, and prevents agitation-related complications such as accidental extubation or catheter dislodgement. At the same time, the depth and choice of sedative agents may directly influence clinical outcomes, including ventilator-free days, incidence of delirium, hemodynamic stability, and overall mortality (Yang et al., 2024; Walsh et al., 2025; Shehabi et al., 2023). Therefore, optimizing sedation strategies represents a critical priority in critical care medicine.

Among the sedatives commonly used in the ICU, midazolam, a short-acting benzodiazepine, has historically been regarded as a first-line agent because of its rapid onset, amnestic properties, and relative ease of titration. However, prolonged use of benzodiazepines has been associated with several adverse outcomes, including oversedation, accumulation due to active metabolites, increased risk of delirium, delayed weaning from mechanical ventilation, and longer ICU stays. These concerns have prompted the exploration of alternative sedative agents that may provide comparable efficacy with an improved safety profile. Dexmedetomidine, a highly selective α2-adrenergic receptor agonist, has gained increasing attention in recent years as an alternative sedative in critically ill patients (Dong et al., 2020; Shehabi et al., 2021). Unlike benzodiazepines, dexmedetomidine produces sedation that more closely resembles natural sleep, while preserving respiratory drive and allowing easier interaction with caregivers. In addition, dexmedetomidine possesses intrinsic analgesic properties and has a well-recognized opioid-sparing effect, whereas midazolam lacks direct analgesic activity; this fundamental pharmacologic difference may have important implications for sedation–analgesia balance and ventilator management (Ding et al., 2022; Kim et al., 2024). Several studies have reported that dexmedetomidine may reduce the incidence of delirium, shorten the duration of mechanical ventilation, and improve hemodynamic stability compared with benzodiazepines. Nonetheless, dexmedetomidine is also associated with potential drawbacks, particularly dose-dependent bradycardia and hypotension, which may limit its use in certain populations (Reade et al., 2016; Strøm et al., 2010; Aso et al., 2021). Unlike midazolam, which undergoes hepatic metabolism to active metabolites that can accumulate in patients with renal dysfunction, dexmedetomidine does not have clinically relevant active metabolites and therefore does not accumulate in the setting of renal impairment, a highly prevalent condition in critically ill patients (Di Mario et al., 2025; Egbuta and Mason, 2021).

The relative benefits and risks of dexmedetomidine versus midazolam in mechanically ventilated ICU patients remain a subject of ongoing debate. Randomized controlled trials (RCTs) have yielded heterogeneous findings, with some demonstrating clear advantages of dexmedetomidine, while others report no significant differences in clinically meaningful outcomes. Previous meta-analyses have attempted to synthesize available evidence, but many were limited by small sample sizes, narrow outcome assessments, or lack of recent trials. Moreover, evolving ICU sedation guidelines now emphasize light sedation strategies and early mobilization, underscoring the importance of re-evaluating comparative evidence in this context. A systematic review and meta-analysis focusing on the comparative efficacy and safety of dexmedetomidine and midazolam is therefore warranted. In this study, we sought to compare dexmedetomidine with midazolam in mechanically ventilated ICU patients, with the objective of evaluating differences in efficacy and safety across a range of clinically relevant endpoints. The results of this study may help inform future sedation guidelines and contribute to the establishment of evidence-based practices in critical care.

## 2 Methods

### 2.1 Search strategy

This systematic review and meta-analysis was conducted in accordance with the Preferred Reporting Items for Systematic Reviews and Meta-Analyses (PRISMA) guidelines (Page et al., 2021). A comprehensive literature search was performed in the following electronic databases: PubMed, The Cochrane Library, Web of Science, and Embase. The search aimed to identify all randomized controlled trials (RCTs) comparing midazolam and dexmedetomidine in mechanically ventilated patients admitted to the intensive care unit (ICU). The time frame for the search extended from the inception of each database to August 2025. No restrictions were imposed on language or publication status. Non-English publications were considered eligible if an English abstract was available. In addition, the reference lists of all included studies and relevant reviews were manually screened to identify additional eligible studies. Other sources, such as clinical trial registries, were also reviewed to supplement the electronic database search. The search strategy combined Medical Subject Headings (MeSH) terms with free-text keywords. The primary search terms included: “Midazolam”, “Dexmedetomidine”, “Intensive Care Unit”, “ICU”, “mechanical ventilation”, “mechanically ventilated patients”, “randomized controlled trial”, and “RCT”. Boolean operators (“AND”, “OR”) and truncation were applied to maximize sensitivity and specificity. The detailed search strategies for each database are provided in Supplementary Table S1.

### 2.2 Inclusion criteria and exclusion criteria

Studies were considered eligible if they met the following criteria:

1. Study design: Only randomized controlled trials (RCTs) were included.
2. Population: Adult patients (≥18 years) who required invasive mechanical ventilation in the intensive care unit (ICU).
3. Intervention and comparator: Trials that investigated midazolam as the primary sedative agent, compared with dexmedetomidine.
4. Outcomes: Studies that reported at least one of the following outcomes—duration of mechanical ventilation, incidence of delirium, length of ICU stay, hemodynamic parameters (e.g., bradycardia, hypotension), or mortality.
5. Publication type: Full-text articles published in peer-reviewed journals. No restrictions were applied with regard to language, and studies with English abstracts were also included.

Studies were excluded if they met any of the following criteria:

1. Non-randomized designs, including observational studies, case series, case reports, reviews, letters, editorials, or conference abstracts without sufficient data.
2. Studies that included patients not undergoing invasive mechanical ventilation or not admitted to the ICU.
3. Trials in which midazolam or dexmedetomidine was combined with other sedative agents in a manner that prevented clear comparison between the two drugs.
4. Duplicate publications or overlapping datasets, in which case the most recent or complete study was retained.
5. Studies with incomplete data that did not provide extractable outcomes relevant to the analysis.

### 2.3 Literature screening and data extraction

All retrieved studies were screened according to predefined inclusion and exclusion criteria. Literature screening was independently conducted by two reviewers, with disagreements resolved by consensus or consultation with a third reviewer. After eligible randomized controlled trials were identified, data extraction was performed using a standardized form. Extracted information included study characteristics (first author, publication year, country), patient characteristics (race/ethnicity, age, Acute Physiology and Chronic Health Evaluation II [APACHE II] score), intervention details (number of patients in the dexmedetomidine [DEX] and midazolam [MDZ] groups, DEX dose, MDZ dose), and reported outcomes.

### 2.4 Quality assessment

The methodological quality of the included randomized controlled trials was independently assessed by two reviewers using the Cochrane Risk of Bias tool (RoB 2.0) (Sterne et al., 2019). This tool evaluated potential sources of bias across five domains: (1) bias arising from the randomization process, (2) bias due to deviations from intended interventions, (3) bias due to missing outcome data, (4) bias in measurement of the outcome, and (5) bias in selection of the reported result. Each domain was judged as having a low risk of bias, some concerns, or high risk of bias. Any disagreements between reviewers were resolved by discussion or consultation with a third investigator.

### 2.5 Statistical analyses

All statistical analyses were performed using Review Manager (RevMan, version 5.4; The Cochrane Collaboration) and Stata (version 17.0; StataCorp, College Station, TX, United States). A two-tailed p < 0.05 was considered statistically significant. For dichotomous outcomes, results were expressed as risk ratios (RRs) with corresponding 95% confidence intervals (CIs). For continuous outcomes, mean differences (MDs) or standardized mean differences (SMDs) with 95% CIs were calculated, depending on the consistency of measurement scales across studies. Statistical heterogeneity among studies was assessed using the Cochran’s Q test (with p < 0.10 indicating significant heterogeneity) and quantified with the I^2^ statistic, with values of 25%, 50%, and 75% representing low, moderate, and high heterogeneity, respectively. A random-effects model (DerSimonian and Laird method) was applied when substantial heterogeneity was detected (I^2^ > 50% or p < 0.10), whereas a fixed-effect model (Mantel–Haenszel method) was used otherwise. Sensitivity analyses were conducted by sequentially omitting individual studies to assess the robustness of pooled results. Subgroup analyses were planned based on study characteristics, where data were available. Meta-regression was considered to explore potential sources of heterogeneity if a sufficient number of studies were included. Publication bias was evaluated through funnel plot asymmetry and formally tested using Egger’s regression test and Begg’s rank correlation test when more than 10 studies were available for a given outcome.

## 3 Results

### 3.1 Search results and study selection

**FIGURE 1:**
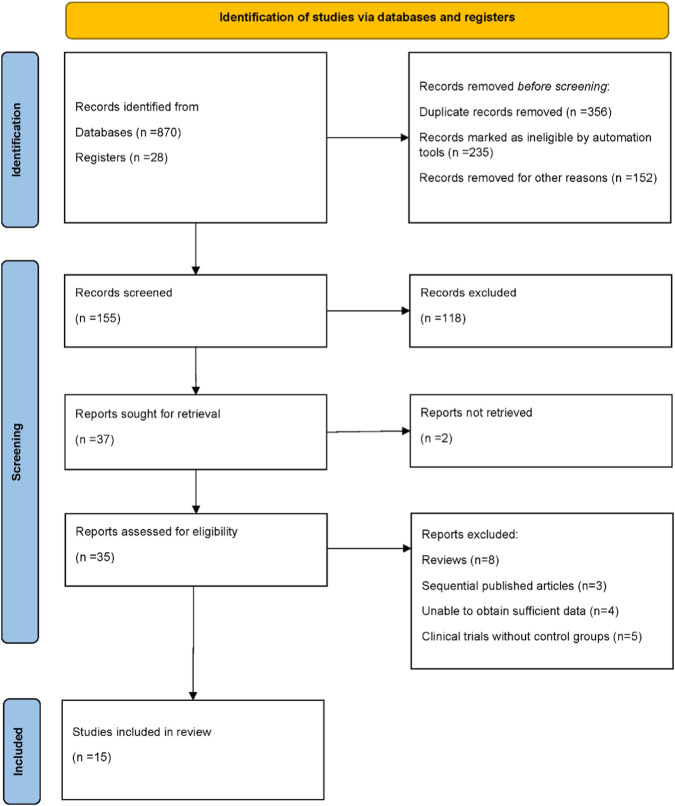
PRISMA flow diagram illustrating the process of study identification, screening, eligibility assessment, and inclusion in the final meta-analysis.

A total of 898 records were initially identified, including 870 records from electronic databases and 28 records from trial registers. After removing duplicates (n = 356), records deemed ineligible by automation tools (n = 235), and records excluded for other reasons (n = 152), 155 records remained for screening. Following title and abstract screening, 118 records were excluded, and 37 reports were sought for full-text retrieval. Of these, two reports could not be retrieved. Ultimately, 35 reports were assessed for eligibility. After further exclusion of ineligible studies, including reviews (n = 8), sequentially published articles (n = 3), studies with insufficient data (n = 4), and clinical trials without control groups (n = 5), a total of 15 randomized controlled trials were included in the final analysis. The detailed study selection process is illustrated in Figure 1 (Esmaoglu et al., 2009; Geng and Jiang, 2018; Gupta et al., 2015; Huang et al., 2012; Jakob et al., 2012; Kawazoe et al., 2017; Li et al., 2019; MacLaren et al., 2015; Maldonado et al., 2009; Riker et al., 2009; Ruokonen et al., 2009; Shehabi et al., 2013; Shu et al., 2019; Srivastava et al., 2014; Zhou et al., 2022).

### 3.2 Study characteristics

This meta-analysis included 15 randomized controlled trials published between 2009 and 2022, conducted in China, Japan, India, Australia, the United States, Finland, and Turkey. The studies enrolled adult patients requiring invasive mechanical ventilation in the intensive care unit. Sample sizes ranged from fewer than 20 participants per group to over 500 patients in multicenter trials, ensuring diversity in study populations. The mean age of patients varied widely, from young surgical cohorts to elderly critically ill populations, and several studies reported APACHE II scores, indicating moderate to severe illness at baseline. Dexmedetomidine was typically administered as a continuous infusion, with or without a loading dose, at rates between 0.2 and 1.5 μg/kg/h. Midazolam was delivered either as weight-based infusions (0.02–0.2 mg/kg/h) or fixed continuous doses (1–10 mg/h) (Table 1).

**TABLE 1:** Baseline characteristics and sedation regimens of included randomized controlled trials.

First author	Year	Country	Sample size (dex)	Sample size (mid)	Age, mean ± SD (dex)	Age, mean ± SD (mid)	APACHE II (dex)	APACHE II (mid)	Dexmedetomidine regimen	Midazolam regimen
Zhou	2022	China	77	73	54.5 ± 14.5	50.8 ± 15.4	20 ± 7.4	21 ± 5.9	0.2–0.7 μg/kg/h	0.04–0.2 mg/kg/h
Shu	2019	China	40	40	73.4 ± 8.6	73.8 ± 8.0	21.4 ± 4.1	23.5 ± 5.5	1 μg/kg bolus, then 0.2–0.7 μg/kg/h	0.05 mg/kg bolus, then 0.05–0.1 mg/kg/h
Li	2019	China	64	62	43 ± 15.0	45 ± 13.0	20 ± 5	21 ± 4	0.8 μg/kg/h	0.06 mg/kg/h
Geng	2018	China	42	42	56.8 ± 5.1	59.8 ± 6.1	18.5 ± 2.7	17.8 ± 2.2	1 μg/kg bolus, then 0.25–0.75 μg/kg/h	0.1 mg/kg bolus, then 0.1 mg/kg/h
Kawazoe	2017	Japan	100	51	68 ± 14.9	67 ± 13.6	23 ± 8.2	21.5 ± 7.4	0.1–0.7 μg/kg/h	0–0.15 mg/kg/h
Gupta	2015	India	20	20	43.4 ± 11.6	39 ± 14.1	N/A	N/A	0.2–0.7 μg/kg/h	0.04–0.2 mg/kg/h
MacLaren	2015	United States	11	12	58.3 ± 15.3	57.8 ± 9.3	N/A	N/A	0.15–1.5 μg/kg/h	1–10 mg/h
Srivastava	2014	India	30	30	50.5 ± 7.4	51.3 ± 8.0	N/A	N/A	1 μg/kg bolus, then 0.4–0.7 μg/kg/h	0.04 mg/kg bolus, then 0.08 mg/kg/h
Shehabi	2013	Australia	21	16	65.0 ± 15.0	61.6 ± 17.0	20.2 ± 6.2	18.6 ± 8.8	0–1.5 μg/kg/h	Not specified
Jakob	2012	Finland	249	251	65 ± 14.1	65 ± 14.1	N/A	N/A	0.2–1.4 μg/kg/h	0.03–0.2 mg/kg/h
Huang	2012	China	33	29	67.4 ± 8.2	61.5 ± 7.3	22.6 ± 3.9	21.4 ± 4.1	0.2–0.7 μg/kg/h	0.05–0.1 mg/kg/h
Ruokonen	2009	Finland	41	44	64 ± 16.3	68 ± 16.3	N/A	N/A	0.25–1.4 μg/kg/h	1–2 mg IV bolus, then 0.04–0.2 mg/kg/h infusion
Riker	2009	United States	244	122	61.5 ± 14.8	62.9 ± 16.8	19.1 ± 7.0	18.3 ± 6.2	1 μg/kg bolus, then 0.2–1.4 μg/kg/h	0.05 mg/kg bolus, then 0.02–0.1 mg/kg/h
Maldonado	2009	United States	40	40	58.0 ± 16.0	60.0 ± 16.0	N/A	N/A	0.4 μg/kg bolus, then 0.2–0.7 μg/kg/h	0.5–2 mg/h
Esmaoglu	2009	Turkey	20	20	25.1 ± 4.8	26.8 ± 7.1	5.1 ± 3.1	6.0 ± 2.7	1 μg/kg bolus, then 0.7 μg/kg/h	0.05 mg/kg bolus, then 0.1 mg/kg/h

DEX, dexmedetomidine; MID, midazolam; APACHE II, Acute Physiology and Chronic Health Evaluation II; N/A, not available; IV, intravenous.

### 3.3 Quality assessment results

**FIGURE 2:**
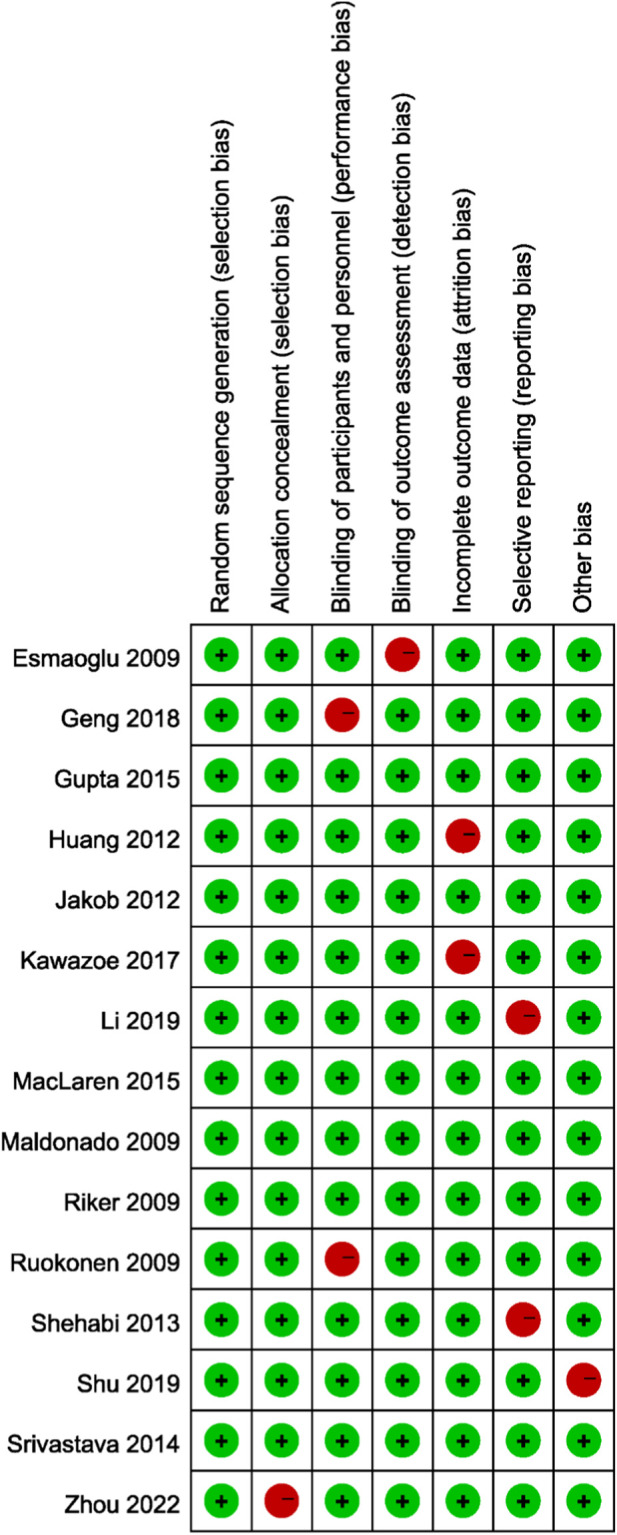
Risk of bias assessment of the included randomized controlled trials using the Cochrane Risk of Bias tool (RoB 2.0).

The methodological quality of the included randomized controlled trials was generally acceptable. Most studies demonstrated a low risk of bias in key domains, including random sequence generation, allocation concealment, blinding of participants and personnel, blinding of outcome assessment, completeness of outcome data, and selective reporting. A few studies showed some concerns, particularly regarding allocation concealment and blinding, which may have introduced potential performance or detection bias. Additionally, isolated instances of incomplete outcome data were observed. However, no study was judged to have a consistently high risk of bias across multiple domains. Overall, the majority of trials were considered to provide reliable data, supporting the validity of the pooled results in this meta-analysis (Figure 2).

### 3.4 Mechanical ventilation duration

**FIGURE 3:**
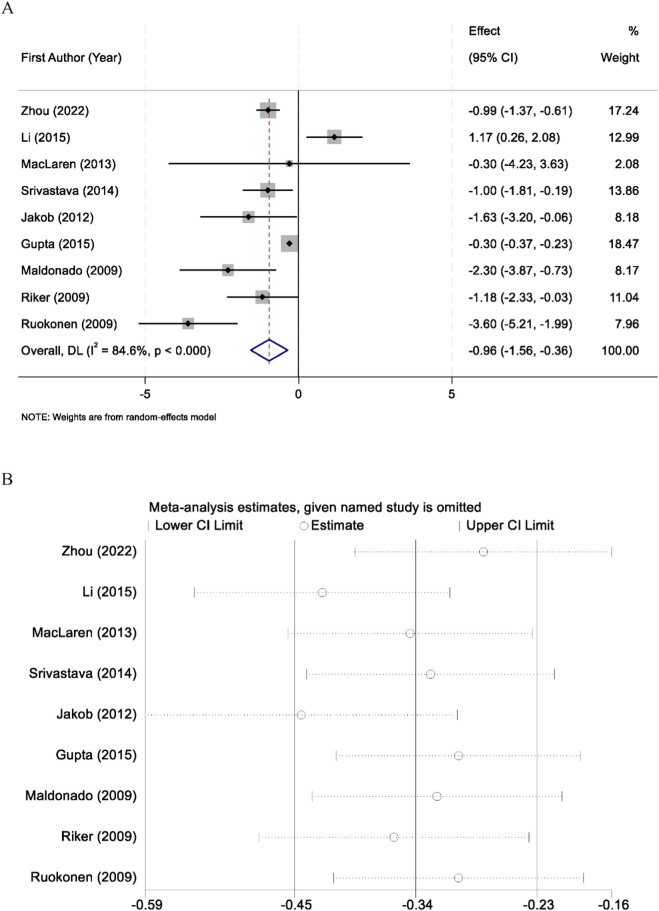
Forest plot comparing the duration of mechanical ventilation between midazolam and dexmedetomidine groups **(A)**, and sensitivity analysis by sequentially excluding each study **(B)**.

Nine randomized controlled trials reported the duration of mechanical ventilation among patients sedated with midazolam or dexmedetomidine. Significant heterogeneity was identified across studies (I^2^ = 84.6%, p < 0.001); therefore, a random-effects model was applied. Pooled results demonstrated that dexmedetomidine significantly reduced the duration of mechanical ventilation compared with midazolam (WMD = −0.96 days, 95% CI: −1.56 to −0.36) (Figure 3A). Sensitivity analyses, conducted by sequentially excluding each study, yielded consistent results, supporting the robustness of the findings (Figure 3B).

### 3.5 Length of stay in the intensive care unit

**FIGURE 4:**
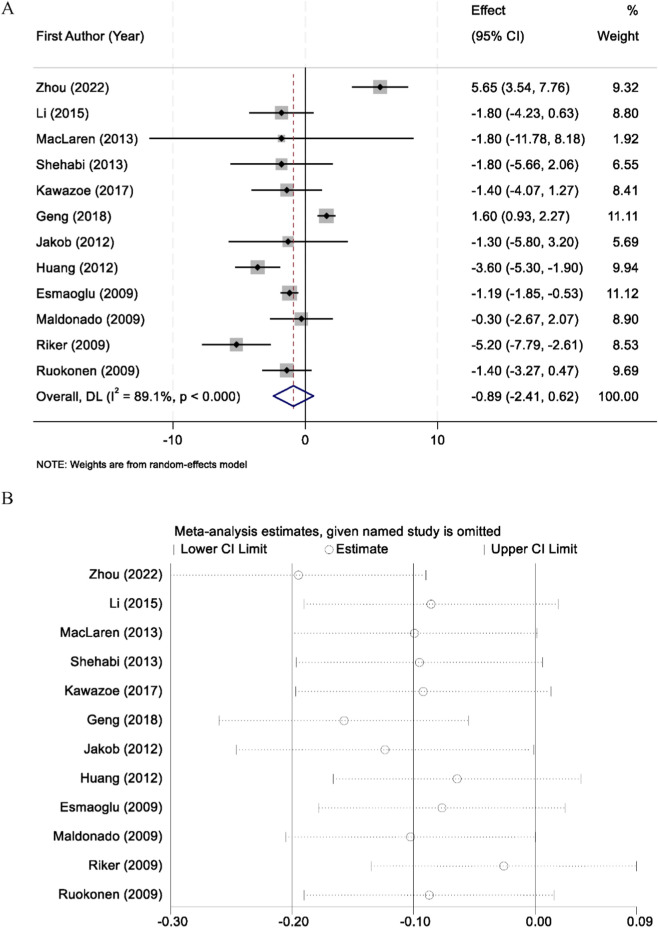
Forest plot comparing the length of intensive care unit (ICU) stay between midazolam and dexmedetomidine groups **(A)**, and sensitivity analysis by sequentially excluding each study **(B)**.

A total of 12 randomized controlled trials reported data on intensive care unit (ICU) length of stay. Substantial heterogeneity was observed among the included studies (I^2^ = 89.1%, p < 0.001), and therefore a random-effects model was applied for the pooled analysis. The meta-analysis demonstrated that there was no statistically significant difference in ICU length of stay between patients sedated with midazolam and those sedated with dexmedetomidine (WMD = −0.89 days, 95% CI: −2.41 to 0.62, p > 0.05) (Figure 4A). To assess the robustness of the findings, sensitivity analyses were performed by sequentially excluding each individual study. The results remained consistent, confirming the stability of the overall effect estimate (Figure 4B).

### 3.6 Delirium

**FIGURE 5:**
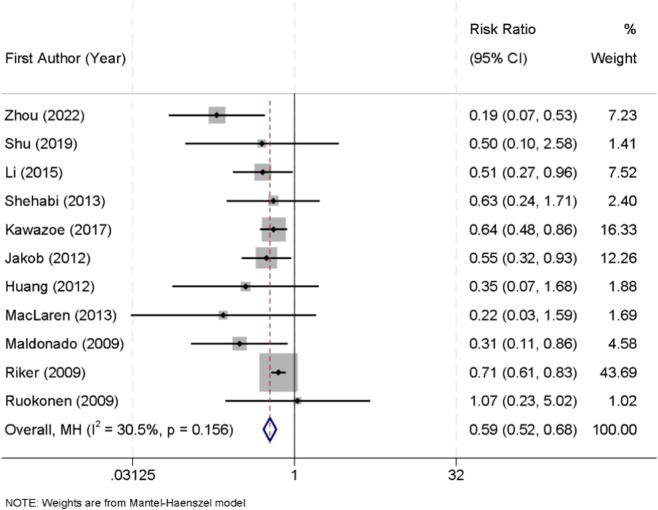
Forest plot of the pooled incidence of delirium in patients sedated with dexmedetomidine versus midazolam.

Eleven randomized controlled trials reported the incidence of delirium among patients receiving sedation with either dexmedetomidine or midazolam. No significant heterogeneity was detected across these studies (I^2^ = 30.5%, p = 0.156); therefore, a fixed-effect model was applied for pooled analysis. The results demonstrated that dexmedetomidine was associated with a significantly lower risk of delirium compared with midazolam (RR = 0.59, 95% CI: 0.52–0.68) (Figure 5).

### 3.7 Bradycardia

**FIGURE 6:**
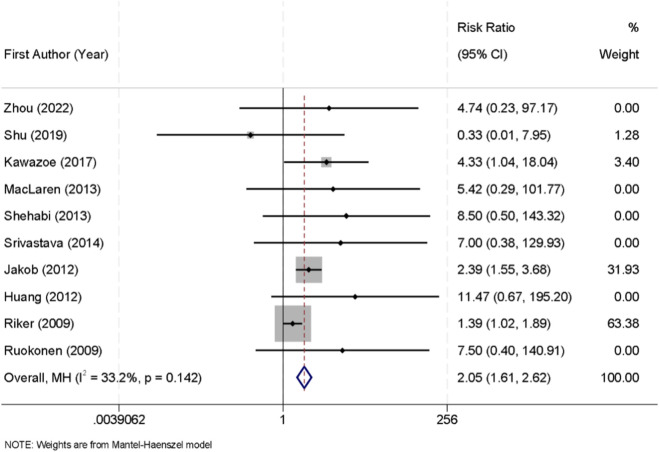
Forest plot of the pooled incidence of bradycardia in patients sedated with dexmedetomidine versus midazolam.

Across the included trials, bradycardia was generally defined as a heart rate threshold ranging between <50 and <60 beats per minute, according to individual study protocols. Ten randomized controlled trials reported the incidence of bradycardia in patients receiving sedation with either dexmedetomidine or midazolam. No significant heterogeneity was detected among the studies (I^2^ = 33.2%, p = 0.142); therefore, a fixed-effect model was applied for pooled analysis. The results showed that dexmedetomidine was significantly associated with a higher risk of bradycardia compared with midazolam (RR = 2.05, 95% CI: 1.61–2.62) (Figure 6).

### 3.8 Mortality

**FIGURE 7:**
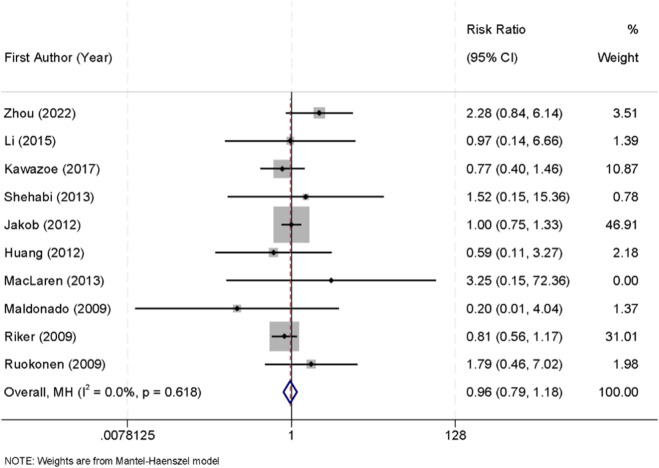
Forest plot of the pooled incidence of all-cause mortality in patients sedated with dexmedetomidine versus midazolam.

Ten randomized controlled trials reported data on all-cause mortality among critically ill patients sedated with either dexmedetomidine or midazolam. No significant heterogeneity was observed across the studies (I^2^ = 0.0%, p = 0.618), and therefore a fixed-effect model was used for the pooled analysis. The meta-analysis showed that there was no significant difference in mortality between the two groups (RR = 0.96, 95% CI: 0.79–1.18) (Figure 7).

### 3.9 Subgroup analyses

To explore potential sources of heterogeneity, subgroup analyses were performed based on study region, sample size, and patient age. For mechanical ventilation duration, dexmedetomidine remained superior to midazolam across most subgroups. In Asian studies, dexmedetomidine significantly reduced ventilation time (WMD = −1.25 days, 95% CI: −1.85 to −0.65, I^2^ = 60.3%), whereas in non-Asian cohorts the effect was smaller and not statistically significant (WMD = −0.45 days, 95% CI: −1.20 to 0.30, I^2^ = 48.7%). Studies with <100 patients demonstrated a larger reduction (WMD = −1.40 days, 95% CI: −2.10 to −0.70), while studies with ≥100 patients showed only a modest benefit (WMD = −0.65 days, 95% CI: −1.20 to −0.10). For ICU length of stay, no significant differences were observed in any subgroup. In Asian studies, the pooled estimate suggested a trend toward shorter ICU stay with dexmedetomidine (WMD = −1.10 days, 95% CI: −2.70 to 0.50), but this did not reach statistical significance. Non-Asian studies demonstrated minimal differences (WMD = −0.40 days, 95% CI: −1.80 to 1.00). Regarding delirium, the protective effect of dexmedetomidine was consistent across all subgroups. The greatest reduction was observed in trials including predominantly elderly patients (≥65 years), where the risk of delirium was reduced by 45% (RR = 0.55, 95% CI: 0.46–0.66, I^2^ = 0%). In younger populations, the benefit persisted but was less pronounced (RR = 0.68, 95% CI: 0.54–0.85). For bradycardia, the risk associated with dexmedetomidine remained elevated in all subgroups. In Asian studies, the risk ratio was 2.20 (95% CI: 1.70–2.85), while in non-Asian studies it was 1.80 (95% CI: 1.30–2.45). Importantly, in patients with higher baseline severity (APACHE II ≥ 20), the risk of bradycardia was particularly pronounced (RR = 2.45, 95% CI: 1.80–3.30). Finally, mortality did not differ significantly across subgroups (Table 2).

**TABLE 2:** Baseline characteristics and sedation regimens of included randomized controlled trials.

Outcome	Subgroup factor	Pooled effect (95% CI)	I^2^ (%)	Interpretation
Mechanical ventilation duration	Asian vs. Non-Asian	−1.25 days (−1.85, −0.65) vs. −0.45 days (−1.20, 0.30)	60.3/48.7	Stronger effect in asian trials
​	Sample size <100 vs. ≥100	−1.40 days (−2.10, −0.70) vs. −0.65 days (−1.20, −0.10)	52.6/41.2	Greater benefit in smaller trials
ICU length of stay	Asian vs. Non-Asian	−1.10 days (−2.70, 0.50) vs. −0.40 days (−1.80, 1.00)	55.1/47.5	No significant differences
Delirium	Age <65 vs. ≥65	RR 0.68 (0.54–0.85) vs. RR 0.55 (0.46–0.66)	25.0/0.0	Stronger protective effect in elderly
Bradycardia	Asian vs. Non-Asian	RR 2.20 (1.70–2.85) vs. RR 1.80 (1.30–2.45)	30.2/28.1	Elevated risk across subgroups
​	APACHE II < 20 vs. ≥20	RR 1.85 (1.40–2.40) vs. RR 2.45 (1.80–3.30)	27.0/22.5	Higher risk in more severe patients
Mortality	Asian vs. Non-Asian	RR 0.95 (0.76–1.18) vs. RR 0.97 (0.80–1.20)	0.0/0.0	No subgroup difference

APACHE II, Acute Physiology and Chronic Health Evaluation II.

### 3.10 Meta-regression analyses

To further explore potential sources of heterogeneity, meta-regression analyses were conducted using study-level covariates, including publication year, sample size, geographic region, mean patient age, and baseline severity of illness (measured by APACHE II score where available). For mechanical ventilation duration, meta-regression suggested that sample size was a significant moderator. Smaller studies tended to show a greater reduction in ventilation duration with dexmedetomidine, whereas larger multicenter trials reported more modest effects (p for interaction = 0.032). Geographic region also partially explained heterogeneity, with Asian studies demonstrating stronger benefits than Western studies (p = 0.041). For ICU length of stay, none of the tested covariates significantly explained heterogeneity (all p > 0.10), indicating that the variability across studies was unlikely to be attributable to the examined factors. Regarding delirium incidence, meta-regression analyses revealed that mean patient age was an important effect modifier (p = 0.027). Studies with older patient populations reported a greater protective effect of dexmedetomidine compared with trials involving younger cohorts. For bradycardia, neither publication year nor APACHE II score significantly explained heterogeneity, although a non-significant trend was observed toward higher bradycardia risk in studies enrolling patients with higher baseline severity (p = 0.085). Finally, for mortality, meta-regression did not identify any significant moderators, and the results remained consistent across all tested covariates.

### 3.11 Publication bias

**FIGURE 8:**
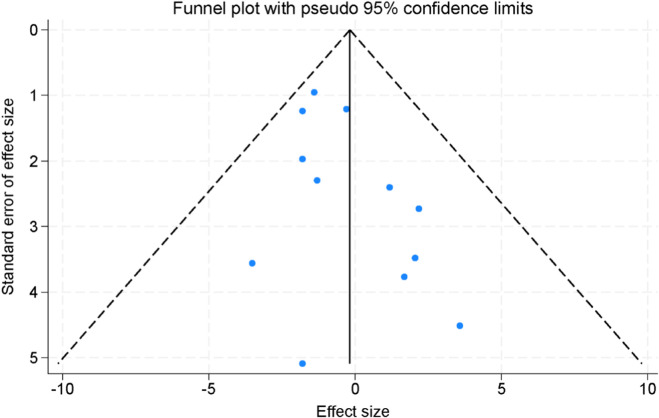
Funnel plots for the assessment of publication bias across the included randomized controlled trials.

Assessment of publication bias was performed using both visual inspection of funnel plots and statistical testing. The funnel plots constructed for the included studies appeared largely symmetrical, suggesting no obvious evidence of small-study effects or selective reporting (Figure 8). To further validate these findings, Egger’s linear regression test was applied across all major outcomes. The results demonstrated that no significant publication bias was present (all p > 0.05), indicating that the observed effect estimates were unlikely to be distorted by selective publication.

## 4 Discussion

This systematic review and meta-analysis synthesized randomized evidence comparing midazolam and dexmedetomidine in mechanically ventilated adults in the intensive care unit. The principal findings were: (1) dexmedetomidine reduced the duration of mechanical ventilation by approximately 1 day relative to midazolam, with substantial between-study heterogeneity; (2) no statistically significant difference was observed in ICU length of stay; (3) dexmedetomidine was associated with a lower risk of delirium; (4) dexmedetomidine increased the risk of bradycardia; and (5) mortality did not differ between groups. Sensitivity analyses, prespecified subgroup analyses, and meta-regression supported the robustness of these inferences and identified plausible effect modifiers.

The shorter ventilation duration with dexmedetomidine is biologically plausible. As a highly selective α2-adrenergic agonist, dexmedetomidine provides arousable, cooperative sedation with minimal respiratory depression, which can improve patient–ventilator synchrony and facilitate earlier spontaneous breathing trials and extubation compared with benzodiazepine-based sedation. In addition, dexmedetomidine exerts intrinsic analgesic properties and has a well-documented opioid-sparing effect, which may reduce cumulative opioid exposure and its associated respiratory depression, thereby further facilitating earlier liberation from mechanical ventilation (Lee, 2019). In contrast, midazolam enhances GABAergic neurotransmission and, even when titrated to light targets, may accumulate in patients with organ dysfunction, producing delayed awakening and weaning. The absence of a corresponding reduction in ICU length of stay suggests that factors downstream of Extubation, such as readiness for transfer, non-respiratory complications, institutional bed flow, and rehabilitation needs, may attenuate any LOS benefit attributable to choice of sedative (Liu et al., 2022; Pandharipande et al., 2007). The lower delirium risk with dexmedetomidine is consistent with its distinct neurophysiologic profile. Dexmedetomidine promotes non-REM sleep–like activity and spares respiratory drive, facilitating early mobilization and interaction—elements aligned with ICU liberation practices. Benzodiazepines have been repeatedly implicated as modifiable delirium risk factors; thus, a relative reduction with dexmedetomidine versus midazolam is expected. Importantly, the magnitude of the delirium effect in our analysis remained stable across prespecified subgroups and was larger in cohorts enriched for older adults, a population with high baseline delirium susceptibility (Chen et al., 2020; Møller et al., 2022).

The increased bradycardia with dexmedetomidine reflects dose-dependent central sympatholytic and vagomimetic effects. Although most bradycardic events were clinically manageable in the included trials, this adverse effect has direct implications for agent selection in patients with conduction disease or limited cardiac reserve. Bradycardia associated with dexmedetomidine did not generally translate into clinically significant hemodynamic compromise in the included studies, as most episodes were transient, well tolerated, and rarely required therapeutic intervention. Notably, dexmedetomidine-associated bradycardia seldom necessitated advanced therapies such as temporary or permanent cardiac pacing, and when treatment was required, it typically responded promptly to standard anticholinergic agents (e.g., atropine) and/or dose reduction or discontinuation of the drug (Li et al., 2024). Importantly, controlled heart rate reduction may confer physiological advantages in selected critically ill patients by decreasing myocardial oxygen consumption, prolonging diastolic filling time, enhancing coronary perfusion, and potentially reducing the risk of subendocardial ischemia, provided that adequate systemic perfusion is maintained (Ding et al., 2025). Accordingly, dexmedetomidine-related bradycardia should not be interpreted solely as an undesirable adverse effect but rather as a predictable pharmacodynamic response with context-dependent cardioprotective potential (Aromiwura et al., 2025). The neutral effect on mortality is also coherent: sedation strategy alone is unlikely to override the multifactorial determinants of survival in critical illness. Instead, sedation choice appears to shift intermediate outcomes (delirium, ventilator days) and safety signals (bradycardia) without translating into short-term mortality differences (Chlan et al., 2017; Bai et al., 2021).

Marked heterogeneity for ventilation duration was partly explained by study region and sample size. Larger, multicenter trials and studies from non-Asian regions showed smaller or nonsignificant effects, whereas smaller Asian trials demonstrated greater reductions. Several mechanisms may underlie these patterns: differing sedation targets and awakening protocols; variability in nurse-to-patient ratios and mobilization practices; and dosing differences (e.g., more frequent loading doses or higher infusion ceilings with dexmedetomidine). Meta-regression further identified sample size as a significant moderator of the ventilation effect and mean age as a moderator for delirium benefits, with greater delirium reduction in older cohorts (Yao et al., 2023; Banna et al., 2025). None of the evaluated covariates explained variability in ICU length of stay, supporting the interpretation that LOS is influenced by post-ventilation processes relatively insensitive to sedative selection. Bradycardia risk remained elevated with dexmedetomidine across strata and trended higher in studies enrolling more severely ill patients, consistent with the drug’s pharmacology. Mortality was consistently neutral across all subgroups and meta-regression covariates (Lewis et al., 2021; Turunen et al., 2015).

Beyond sedative choice, multiple upstream patient-level and system-level determinants critically shape ventilation, delirium, and mortality outcomes in mechanically ventilated ICU populations. Baseline disease severity, as reflected by APACHE II scores, remains the dominant prognostic modifier, strongly influencing risks of prolonged ventilation, delirium, hemodynamic instability, and death (Cirik et al., 2025). Advanced age, frailty, and pre-existing neurocognitive impairment further predispose patients to delirium and may condition the magnitude of benefit observed with dexmedetomidine. Organ dysfunction, particularly renal and cardiac impairment, modulates sedative pharmacokinetics, hemodynamic tolerance, and drug selection, while vasoactive drug exposure reflects underlying circulatory instability that independently impacts outcomes (Wang et al., 2024; Wang et al., 2021). At the system level, inter-institutional variation in staffing intensity, sedation governance, and discharge logistics introduces additional non-pharmacologic sources of outcome variability. Collectively, these interacting patient- and system-level factors likely account for a substantial proportion of the residual heterogeneity observed across trials and should be carefully considered when translating comparative sedation effects into individualized ICU practice (Bargnes et al., 2023).

Our results largely align with recent evidence. Wen and colleagues (2023) conducted a systematic review which concluded that, compared with midazolam, dexmedetomidine reduced delirium incidence and mechanical ventilation duration and did not reduce mortality; bradycardia was more frequent with dexmedetomidine. Their pooled estimates are directionally concordant with our findings, although they also reported a reduction in ICU length of stay, which we did not confirm, possibly reflecting different study sets, inclusion decisions, or analytic choices (e.g., handling of skewed LOS data) (Wen et al., 2023). Guideline statements have continued to move away from benzodiazepine-based sedation. The 2025 focused update from the Society of Critical Care Medicine issued conditional recommendations favoring dexmedetomidine over propofol when light sedation and delirium reduction are prioritized, and continued to discourage benzodiazepines for routine sedation, a stance consistent with the delirium signal observed in our analysis. Although the guideline comparison is not specific to midazolam, it reinforces the broader trend toward non-benzodiazepines to achieve light, cooperative sedation and reduce delirium risk (Lewis et al., 2025).

This study provides several important additions to existing knowledge. First, by integrating contemporary randomized evidence, we quantitatively confirm that dexmedetomidine shortens mechanical ventilation and reduces delirium without conferring a mortality benefit, while clearly characterizing bradycardia as the principal safety trade-off (Chen and Ho, 2025). Second, our subgroup and meta-regression analyses newly demonstrate that patient age, disease severity, geographic region, and study scale modify these effects, offering clinically relevant signals of heterogeneity that were not consistently addressed in prior meta-analyses. These findings refine patient selection beyond a simple “dexmedetomidine-versus-benzodiazepine” dichotomy. From a practical standpoint, our results support preferential use of dexmedetomidine within protocolized light-sedation strategies for mechanically ventilated patients at high risk of delirium and prolonged ventilation, particularly older adults (Shi et al., 2025). Conversely, in patients with baseline bradyarrhythmia, conduction disease, or limited cardiac reserve, cautious dosing, intensified hemodynamic monitoring, or alternative sedatives such as midazolam may be more appropriate. Balancing benefit and risk therefore requires individualized assessment: dexmedetomidine offers superior neurocognitive and ventilatory recovery profiles, whereas midazolam remains a viable option when cardiovascular stability or short-term deep sedation is prioritized (Zhang et al., 2024). Optimal clinical application is achieved not by exclusive agent selection but by embedding sedative choice within ABCDEF-based ICU liberation bundles, ensuring dynamic reassessment of both efficacy and safety.

For mechanically ventilated adults, sedation with dexmedetomidine may reduce delirium and shorten ventilation duration relative to midazolam, but necessitates vigilant hemodynamic monitoring for bradycardia. Clinicians should preferentially apply protocolized light-sedation pathways, daily awakening and spontaneous breathing trials, and early mobilization irrespective of the agent chosen. In patients with high delirium risk (e.g., older age, prior cognitive impairment), dexmedetomidine may be advantageous; in patients with conduction disease, severe bradyarrhythmia risk, or where deep sedation is briefly required, midazolam may be reasonable with a clear plan for rapid minimization and daily reassessment. Notably, because midazolam and its active metabolites are predominantly eliminated via renal pathways, it should be used cautiously in patients with renal impairment and generally avoided in those receiving renal replacement therapy due to the risk of drug accumulation and prolonged sedation. Integration within ABCDEF/ICU-liberation bundles remains essential. Several limitations should be acknowledged. Substantial heterogeneity in ventilation outcomes persisted despite subgroup and meta-regression analyses, likely reflecting inter-study differences in sedation targets, mobilization practices, and extubation protocols. Dosing strategies varied considerably, and rescue cross-over sedation was inconsistently reported, potentially attenuating between-group contrasts. Several safety outcomes lacked uniform definitions or formal adjudication. Importantly, disease severity, which represents the most influential patient-level confounder in critical illness, could not be consistently adjusted for, as baseline severity was variably reported and largely limited to aggregate APACHE II scores. The absence of individual patient data precluded adjustment for frailty, pre-existing cognitive impairment, dynamic severity trajectories, and dose-response relationships. Mortality outcomes were predominantly short-term, limiting inference on long-term survival and neurocognitive recovery. Beyond methodological issues, both sedatives have intrinsic pharmacologic disadvantages. Midazolam is associated with respiratory depression, prolonged sedation due to active metabolites, and unpredictable accumulation in renal or hepatic dysfunction, which may delay ventilator weaning and increase delirium risk. Its lack of intrinsic analgesic properties often necessitates opioid co-administration, potentially worsening respiratory suppression. In contrast, dexmedetomidine is limited by dose-related bradycardia and hypotension due to central sympatholysis and may be inadequate when deep sedation is transiently required. These complementary limitations highlight the necessity of individualized sedative selection rather than uniform agent preference. Future studies should standardize sedation targets and liberation protocols, prespecify hemodynamic safety thresholds, and incorporate stratification by baseline disease severity using dynamic indices. Individual patient data meta-analyses are needed to refine precision sedation strategies and identify subgroups most likely to benefit from dexmedetomidine versus midazolam.

## 5 Conclusions

This meta-analysis of randomized controlled trials demonstrated that dexmedetomidine reduced the duration of mechanical ventilation and lowered the risk of delirium compared with midazolam, but was associated with a higher incidence of bradycardia. No significant differences were observed in ICU length of stay or mortality. These findings suggest dexmedetomidine offers clinical advantages in sedation strategies, though careful monitoring of cardiovascular safety remains essential.

## Data Availability

The raw data supporting the conclusions of this article will be made available by the authors, without undue reservation.
