# Supplementary material for: Efficacy and safety of midazolam versus dexmedetomidine in mechanically ventilated intensive care unit patients: a systematic review and meta-analysis
Source: Front Pharmacol. 2026 Jan 28;17:1733161. doi: 10.3389/fphar.2026.1733161 (PMC12891203; doi:10.3389/fphar.2026.1733161)
Supplement: Supplementary file 1 [file Table1.docx]

**Supplementary Table 1.** Search Strategy.

| **Database** | **Search Strategy** |
| --- | --- |
| PubMed | (((“Midazolam”[Mesh] OR midazolam[tiab]) AND (“Dexmedetomidine”[Mesh] OR dexmedetomidine[tiab])) AND (“Intensive Care Units”[Mesh] OR “ICU”[tiab] OR “critical care”[tiab])) AND (“Respiration, Artificial”[Mesh] OR “Mechanical Ventilation”[tiab] OR “ventilated patients”[tiab]) AND (“Randomized Controlled Trial”[Publication Type] OR randomized[tiab] OR randomised[tiab] OR “RCT”[tiab])) NOT (animals[mh] NOT humans[mh]) |
| Embase | (‘midazolam’/exp OR midazolam:ti,ab) AND (‘dexmedetomidine’/exp OR dexmedetomidine:ti,ab) AND (‘intensive care unit’/exp OR ‘intensive care’:ti,ab OR ICU:ti,ab OR ‘critical care’:ti,ab) AND (‘mechanical ventilation’/exp OR ‘mechanically ventilated patient’:ti,ab OR ‘artificial respiration’:ti,ab) AND (‘randomized controlled trial’/exp OR random*:ti,ab OR RCT:ti,ab) NOT ([animals]/lim NOT [humans]/lim) |
| Web of Science | TS = ((midazolam OR dexmedetomidine) AND (“intensive care unit” OR ICU OR “critical care”) AND (“mechanical ventilation” OR “ventilated patients” OR “artificial respiration”) AND (“randomized controlled trial” OR randomized OR randomised OR RCT)) |
| Cochrane Library | (midazolam:ti,ab,kw OR “Midazolam”[Mesh]) AND (dexmedetomidine:ti,ab,kw OR “Dexmedetomidine”[Mesh]) AND (“Intensive Care Unit”:ti,ab,kw OR ICU:ti,ab,kw OR “critical care”:ti,ab,kw) AND (“Mechanical Ventilation”:ti,ab,kw OR “Artificial Respiration”:ti,ab,kw) AND (“randomized controlled trial”:pt OR randomized:ti,ab,kw OR randomised:ti,ab,kw OR RCT:ti,ab,kw) |
